# The role of spatial frequencies for facial pain categorization

**DOI:** 10.1038/s41598-021-93776-7

**Published:** 2021-07-13

**Authors:** Isabelle Charbonneau, Joël Guérette, Stéphanie Cormier, Caroline Blais, Guillaume Lalonde-Beaudoin, Fraser W. Smith, Daniel Fiset

**Affiliations:** 1 Département de Psychoéducation et de Psychologie, Université du Québec en Outaouais, Gatineau, QC J8X3X7 Canada; 2 University of East Anglia School of Psychology, Norwich, NR4 7TJ UK

**Keywords:** Psychology, Human behaviour

## Abstract

Studies on low-level visual information underlying pain categorization have led to inconsistent findings. Some show an advantage for low spatial frequency information (SFs) and others a preponderance of mid SFs. This study aims to clarify this gap in knowledge since these results have different theoretical and practical implications, such as how far away an observer can be in order to categorize pain. This study addresses this question by using two complementary methods: a data-driven method without a priori expectations about the most useful SFs for pain recognition and a more ecological method that simulates the distance of stimuli presentation. We reveal a broad range of important SFs for pain recognition starting from low to relatively high SFs and showed that performance is optimal in a short to medium distance (1.2–4.8 m) but declines significantly when mid SFs are no longer available. This study reconciles previous results that show an advantage of LSFs over HSFs when using arbitrary cutoffs, but above all reveal the prominent role of mid-SFs for pain recognition across two complementary experimental tasks.

## Introduction

Pain is a subjective experience communicated to others to alert them of potential threats or to seek assistance^1,2^. Effective communication of pain can operate through verbal and non-verbal cues. Among the non-verbal cues, facial expression is considered one of the most reliable indicators of pain^3–5^. The effective recognition of facial expressions of pain is of utmost importance, particularly in nonverbal populations such as infants^6,7^ and adults with dementia^8,9^. However, for its communicative function to be fulfilled, the observer must accurately decode the facial expression depicted.

**Figure 1:**
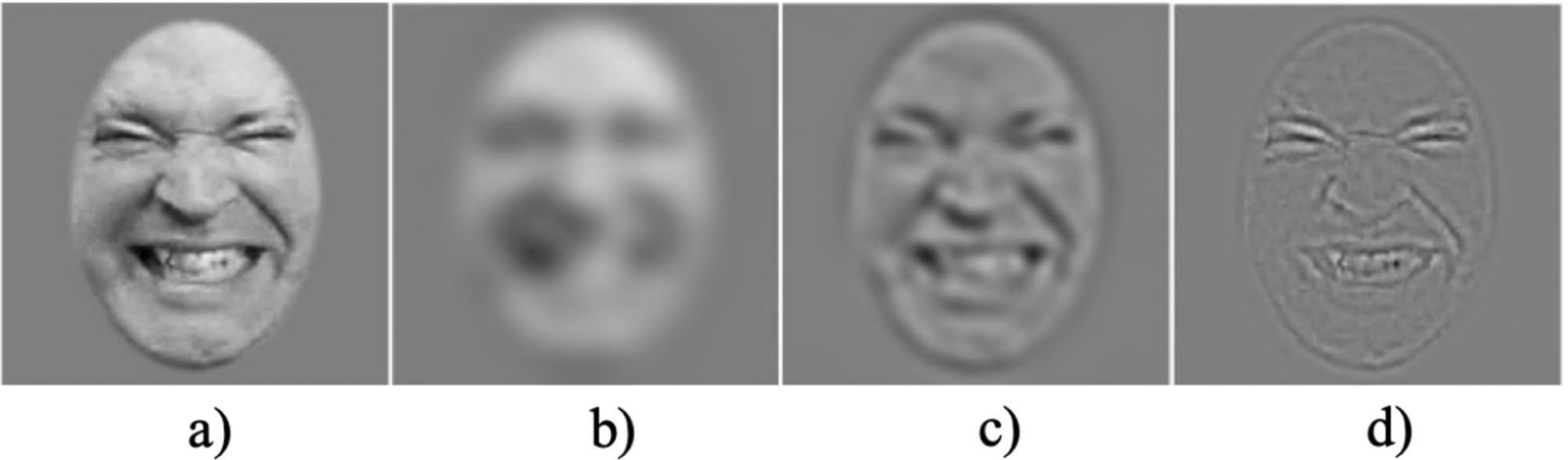
Example of stimuli filtered with a second-order butterworth filter. Note that the vast majority of studies investigating spatial frequencies and facial expressions have employed this filtering method which is different from the Bubble’s method use in this study. Panel (**a**) represents a broadband stimulus, (**b**) a low-pass stimulus (< 8 cpf), (**c**) a band-pass stimulus (between 8 and 32 cpf) and (**d**) a high-pass stimulus (> 32 cpf). MSF as in (**c**) are typically not included in experiments conducted on facial expression processing, including those about pain.

**Figure 2:**
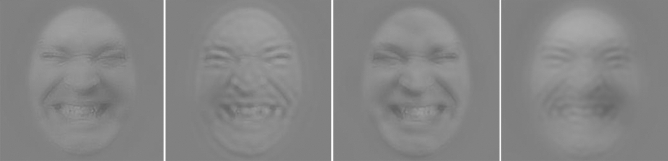
Example of stimuli filtered with the Bubble’s method (see the “Methods” section and Fig. 5 for more details about stimuli creation).

Studies investigating how pain is encoded in facial expressions have highlighted the occurrence of three features: brow furrowing, the wrinkling of the nose with the raising of the upper lip, and the narrowing of the eyes^10–12^. The decoding of pain facial expressions relies on the processing of these features^13,14^. Thus far, the study of low-level visual information underlying this processing has led to inconsistent findings. One of the first steps in vision concerns the decomposition of the visual signal in different spatial frequency (SF) bands^15^. Low SFs (LSFs; see Fig. 1) convey the coarse structures used when viewing faces from a distance^16^ or in periphery^17^ whereas high SFs (HSFs) convey edges and fine details available when faces are viewed from closer and at the fovea^18^. Two recent articles suggest that LSFs play a central role in the recognition of facial expressions of pain^19,20^. However, as a communication signal, one would expect that pain signal would be best suited to a short-to-medium distance where one can benefit from immediate assistance. Furthermore, our previous work using the Bubbles method suggests that no information in lower SF bands (e.g. under 11 cycles per face; cpf) was used to accurately categorize pain^14,21^. Indeed, the results suggest that accurate recognition of pain facial expression relied mostly on SFs between 11 and 85 cpf (for the frown lines region), between 21 and 42 cpf (for the corners of the mouth) and between 11 and 21 cpf (for the entire mouth)^14^. According to the terminology used in previous research^19^ and explained in more details below, these SFs would correspond to mid SFs (MSFs) and high SFs (HSFs). However, one of the criticisms, although never proven, of the Bubbles method is that it modifies the usual perceptual strategy of human observers^22^ and tends to minimize the importance of LSFs. Although previous research does not support the idea that the original version of Bubbles impact perceptual strategies^23^, it is still important to address this issue by using a variant of the Bubbles method (i.e., the SF Bubbles method) which randomly manipulates the presence of SFs in a stimulus (i.e. a face) without hiding some facial regions (see Fig. 2 for stimuli examples). Although results from Roy and colleagues^14,21^ are relevant, it can only isolate the contribution of specific SF bands (e.g. between 42–85, 21–42, 11–21, 5–11 and 3–5 cpf). In this way, the results in terms of SFs are somewhat less precise and could hide part of the phenomenon. For example, one could imagine a scenario in which a SF band is statistically significant for pain recognition although only a portion of the SFs contained in the band are actually useful (e.g. SFs between 11 and 16 in the 11–21 cpf band).

One potential explanation for this discrepancy could lie with Wang and collaborators’^19^ use of cutoffs to isolate the impact of LSF and HSF. In the literature, the cutoffs used to define LSF and HSF vary arbitrarily (e.g. LSF defined below 8 cpf in^19^; between 2 and 8 cpf in^22^; below 6 cycles per image in^24^; and HSF defined above 32 cpf in^19,25^ or above 24 cycles per image^24^). To the best of our knowledge, such variation is not theoretically driven and is often informed by methodological issues (e.g.^26,27^) or for replication purposes^28^. Furthermore, the use of such cutoffs hinders the potential contribution of MSFs, leading to an incomplete or incorrect account of the role of SF in pain perception.

The objective of this study is to offer a more complete account of the role of SF in pain perception by incorporating findings from two different methods. Therefore, experiment 1 aimed to reveal which SFs are the most useful for pain categorization among other emotional expressions (i.e. anger, disgust, fear, joy, sadness, surprise, neutral or pain). This kind of experiment is standard in the facial expression literature (e.g.^14,19^) but instead of using cutoffs to create low-pass and high-pass filters^19^, we used SF Bubbles. The fundamental basis of the Bubbles method and its variants (e.g. SF Bubbles) is that it allows the random sampling of information (e.g. local image features or SFs see Refs.^29–37^) contained in a visual stimulus in order to reveal the relative importance of this information for efficient visual processing. Here in the SF Bubbles method, SFs contained in facial expression images were randomly sampled on each trial (see the “Methods” section for more details on the stimuli creation procedure), allowing to calculate the probability that participants will accurately identify the facial expression presented based on the presence or absence of certain SFs. Therefore, if the sampled SFs are useful for processing a particular facial expression, it will increase the likelihood that participants will respond accurately, and conversely, if they are not useful, it decreases the likelihood that participants will respond accurately.

Experiment 2 aimed to reveal the optimal SFs for pain recognition through the manipulation of the face retinal size (equivalent to the distance between the stimulus and the observer). As in experiment 1, participants were asked to categorize the perceived facial expressions as corresponding to anger, disgust, fear, joy, sadness, surprise, neutral or pain although face images were presented in different sizes. The objective of this experiment was to verify the impact of distance on the ability to categorize the facial expression of pain. Since layers of HSFs are progressively filtered out by increasing distance between the observer and the distal stimulus, this experimental manipulation also allows to investigate the role of relatively high SFs in the presence of lower SFs. This method is also considered more ecological since in everyday life, the distance at which one sees people’s facial expressions can vary considerably.

## Results

Although both of the following experiments included all of the six basic emotions and neutral, only data related to pain will be presented since this article focuses on pain perception in faces (see “Data availability” for access to datasets).

### Experiment 1: SFs for pain categorization

**Figure 3:**
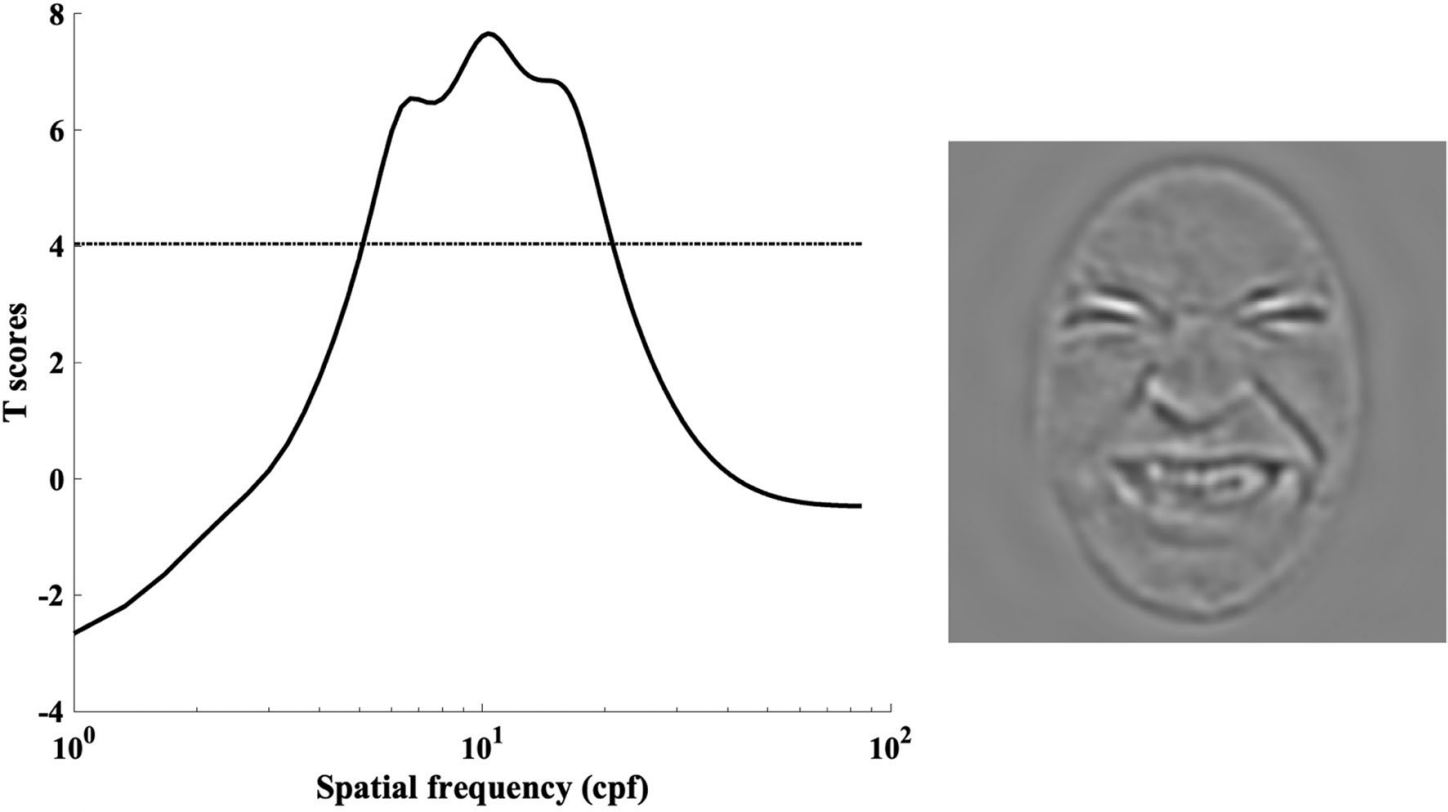
Pain categorization revealed by the Bubble’s method. The left panel displays the SF tuning for pain categorization. The black dotted line represents the statistical threshold for significance (*p* < .05). The right panel represents an example of a stimulus filtered with the significant SFs associated with pain categorization.

Spatial frequencies for accurate pain categorization were analyzed by producing classification images (CI) which represent how strongly each SF is associated with accuracy. This analysis amounts to a multiple regression analysis on the SF filters and accuracies across trials. More specifically, a weighted sum of SF filters was calculated by allocating positive weights to filters that led to correct responses and negative weights to incorrect responses. The idea behind this procedure is that it assumes that bubbles filters that led to correct answers are useful for pain recognition whereas bubbles filters that led to incorrect responses are not relevant for this task. The values of the weights were calculated by transforming raw accuracy on each trial (i.e. zeros and ones) into z scores using the mean and standard deviation of the participant’s accuracies. Z-scoring is intended to minimize the interindividual variability and to control for the actual performance of participants since although the performance level is set at 75%, there can be slight variations in performance related to Quest. Subsequently, the weighted SF filters were transformed into z scores using the expected mean and standard deviation of the null hypothesis given by the Stat4Ci toolbox^38^. Then, to assess the exact contribution of each SF, one-sample t-tests were conducted for each SF using a statistical threshold obtained by the pixel test from the Stat4Ci. The pixel test allows to identify at which t-score the contribution of a particular spatial frequency is considered statistically significant which in this case corresponds to *p* < .05; *t*_crit_ = 4.04 (see the dashed line in Fig. 3). This test compensates for the multiple comparisons across SF, while taking into account the fact that adjacent SF are not totally independent from one another. We also measured the SF peaks by submitting the classification vector to a 50% area SF measure (ASFM; analogous to a 50% area latency measure commonly used in electroencephalography analysis; see^39^). The ASFM corresponds to the SF point that splits the area under the curve and above the significance threshold in two equal parts. Moreover, to verify whether we replicate past observation of an advantage of LSF over HSF, we compared the usefulness of LSF and HSF in our data. For this, we used a bootstrap procedure in which 10,000 resampled classification vectors (in *t* scores) were first produced. Then, in each of these classification vectors, we selected the highest t-score value reached among the SFs below 8 cpf, and the highest t-score value among the SF over or equal to 32 cpf. These values were then compared to one another.

Figure 3 shows the SF tuning for categorizing pain. More precisely, information between 4.5 and 48.4 cpf was significantly and positively associated with accurate pain categorization. The SF most correlated with performance corresponded to 6 cpf. However, given the atypical shape of the frequency tuning curve (typical SF tuning curves usually look more Gaussian; see^36^), we also measured the ASFM which was found at 11.67 cpf. Interestingly, the atypical appearance of the curve could possibly indicate the existence of two distinct peaks, the first reaching its maximal value at 6 cpf (from 4.5 to ~ 14.6 cpf) and the second reaching its maximal value at 22.7 cpf (from ~ 14.6 to 48.4 cpf). Moreover, the present data are not necessarily inconsistent with previous studies^19^. The bootstrap procedure revealed that LSFs were more useful than HSFs for correct categorization of pain in 9961 out of the 10,000 classification vectors (*p* = .0039).

It is also interesting to examine how pain can be miscategorized with other emotions in order to better characterize participants’ performance. The confusability matrix across the six basic emotions as well as pain and neutrality is presented in Table 1. Finally, as a point of comparison, the confusability matrices obtained in Wang’s^19^ and Roy’s^14^ studies (see Table 1 for both studies) were compared with our own. After verifying for data normality, Spearman correlations were calculated and are presented in Table 2. It is interesting to note that in all three studies, the facial expression of pain is systematically confused with disgust and sadness. Furthermore, both happiness and sadness are consistently confused with pain. The only inconsistency between the three studies lies in confusions between pain and fear in Wang’s study^19^. Finally, in regard to other emotions, confusability matrices are highly similar. Confusions are found between anger and disgust, fear and surprise, happiness and neutrality as well as neutrality and sadness^14,19^.

**Table 1:** Confusability matrix depicting the proportion of responses (columns) for each target emotion presented (rows).

Emotion perceived	Emotion presented							
	Pain	Disgust	Fear	Happy	Neutral	Anger	Sadness	Surprise
Pain	**0.5680 (0.3199)**	0.0925	0.0394	0.1265	0.0230	0.0399	0.0787	0.0320
Disgust	0.1523	**0.4884 (0.2633)**	0.0386	0.0361	0.0528	0.1313	0.0699	0.0307
Fear	0.0325	0.0554	**0.5754 (0.3066)**	0.0333	0.0393	0.0381	0.0676	0.1585
Happy	0.0739	0.0434	0.0517	**0.6163 (0.3799)**	0.0786	0.0338	0.0496	0.0527
Neutral	0.0324	0.0532	0.0503	0.0743	**0.5292 (0.2541)**	0.0288	0.1405	0.0913
Anger	0.0438	0.0953	0.0423	0.0474	0.1190	**0.5761 (0.3725)**	0.0361	0.0400
Sadness	0.0816	0.0509	0.0390	0.0379	0.2020	0.0247	**0.5153 (0.2688)**	0.0486
Surprise	0.0239	0.0269	0.2430	0.0280	0.0583	0.0180	0.0301	**0.5680 (0.3189)**

Hits are presented in the diagonal in bold, unbiased hits between parentheses, while omissions (rows—regular font) and false alarms (columns—regular font) are reported for each emotion in the rest of the matrix.

**Table 2:** Spearman correlations of confusability matrices.

Study	*M*	*SD*	Roy et al.^14^	Wang et al.^19^
Roy et al.^14^	0.13	0.20		
Wang et al.^19^	0.12	0.26	.97**	
			[.96, .98]	
Current study	0.12	0.17	.97**	.97**
			[.96, .98]	[.95, .98]

Correlations with 95% confidence intervals in square brackets.

*M* and *SD* are used to represent mean and standard deviation, respectively.

**Indicates *p* < .01.

### Experiment 2: distance for pain categorization

**Figure 4:**
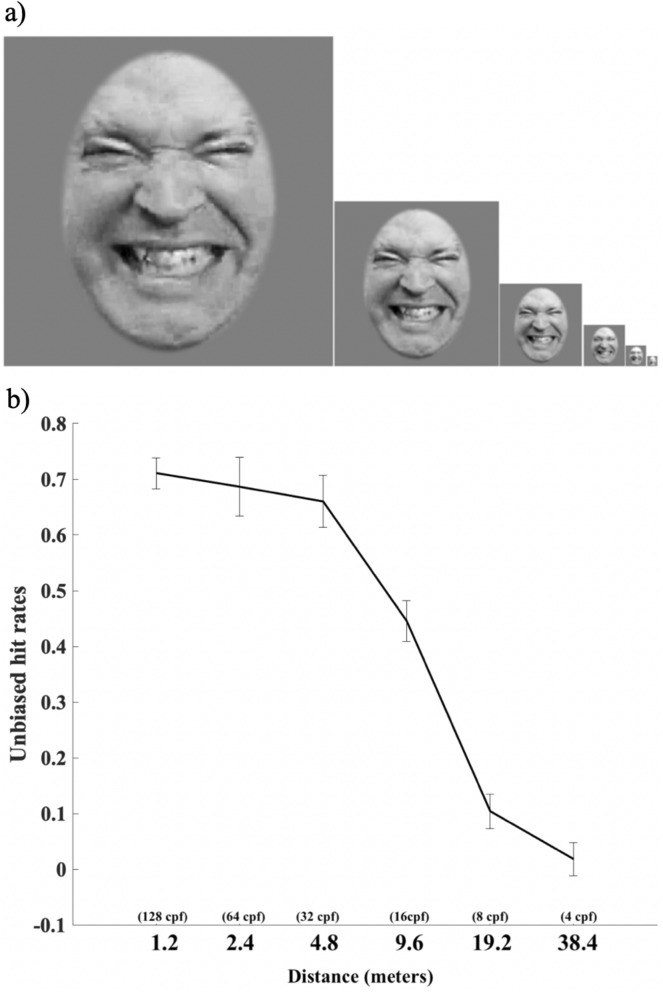
Stimuli and distance for pain categorization. Panel (**a**) Faces stimuli were created with the Laplacian Pyramid toolbox^4^ simulating increasing viewing distances (i.e. images from left to right represent 3.26, 1.63, 0.815, 0.41, 0.20, 0.10 degree of visual angle or a simulated distance of 1.2, 2.4, 4.8, 9.6, 19.2, and 38.4 m). Panel (**b**) Unbiased hit rates for pain categorization as a function of viewing distance. The equivalence in cycles per face (cpf) is presented in parenthesis. Error bars represent the standard error.

To quantify the participants’ performance on the categorization task, unbiased hit rates (see^40^ for details) were computed. This measure refers to a modified form of the signal detection sensitivity measure *d*′ and it allows to quantify sensitivity independently of response bias when discriminating a given expression from the remaining expressions. Note that confusability matrices (with hits and unbiased hit rates) for experiment two for each of the distances are available in the Supplementary Information section. In multiple choice facial expression recognition tasks, unbiased hit rates are usually advised since they can overcome potential biases^41^. For instance, a participant may systematically indicate that he perceives fear when presented with both fear and surprise facial expressions. As a result, his raw scores for fear would be excellent, but he would fail to discriminate fear from surprise. A repeated measures ANOVA on the factor of distance revealed a significant main effect *F*(3.33, 63.33) = 337.27, *p* < .001 (*η*^2^ = 0.95) (see Fig. 4). Post hoc comparisons corrected with Bonferroni’s method revealed significant differences between the various distances. The three conditions representing the closest simulated distances (1.2, 2.4 and 4.8 m) were not significantly different from each other (all *p*’s > .45, all Cohen’s d < 0.41). However, a significant decrease in performance was observed between 4.8 m and 9.6 m (*p* < .001, Cohen’s d = 1.57). Subsequently, all conditions representing the furthest simulated distances (9.6, 19.2 and 38.4 m) showed a large decrease in performance and were all significantly different from each other (all *p*’s < .01, all Cohen’s d > 1.57). Since the pyramid toolbox removes one octave of SF information for each iteration, it is possible to infer the relationship between distance and SFs (i.e. the further away an image is, the more HSFs are lost and only the LSFs remain; see the “Methods” section for details). The results suggest that removing HSFs (over 32 cpf) does not significantly hinder pain categorization. Thereby, when only the high and very high HSFs are removed (i.e. between 128, 64 and 32 cpf), performance for pain recognition is accurate and not significantly different. In other words, it means that an image containing 32 cpf would be as well recognized as an image containing 128 cpf (which contains more HSFs). On the other hand, it is clear that removing MSFs between 16 and 32 cpf and between 8 and 16 cpf significantly decreases performance. The relevance of the results lies in the fact that we see a large decrease in performance when the MSFs are no longer available (i.e. between 32 and 16 cpf as well as between 16 and 8 cpf). As mentioned before, only data regarding pain facial expressions are presented but data on other facial expressions (i.e. anger, disgust, fear, joy, sadness, surprise and neutral) carefully replicate Smith and Schyns (2009) study^16^ despite using different stimuli and participants.

## Discussion

In psychophysics, visibility is a complex concept that refers to the interaction between the properties of the visual system and the characteristics of the distal stimulus. The objective of this paper is to better understand the role of one of these properties. Our argument is that visibility is a function of the availability of certain spatial frequencies and that this availability can be altered either by filtering (Exp. 1) or by increasing the distance between the stimulus and the observer (Exp. 2). Recently, studies on spatial frequency information underlying the processing of pain facial expressions have led to inconsistent findings. While some show an advantage for LSF over HSF^19,20^ others find a preponderance of MSF^14^. With a concern for the generalizability of our findings to more ecological conditions, we verified the impact of distance on the ability to categorize the facial expression of pain. More specifically, our results highlight the importance of a wide range of SFs from LSF (4.5 cpf) to relatively HSF (48.4 cpf) when categorizing pain among other basic emotions. Interestingly, the data presented in experiment 1 may suggest the presence of two peaks that could be linked with facial feature processing. Taken in combination with the data presented by Roy et al.^14,21^ it is possible to interpret the first peak as related to the processing of the mouth area in low-to-mid SF, while the second peak could be associated with the frown line in mid-to-high SF. Of course, this interpretation of our data remains speculative and needs to be taken with caution. It is also important to keep in mind that the size of the smoothing windows used in analysis could have influenced the appearance of the tuning curve. That is, a larger window size might have revealed only one peak instead of two. However, note that in the experiment the smoothing window corresponded to 1.8 cpi and both peaks were still present when the data were analyzed with smoothing windows up to 2.5 cpi.

Confusability matrices across the six basic emotions as well as pain and neutrality revealed a systematic confusion of pain with disgust and sadness that were also found in previous work using the same stimuli^14,19^. It is important to note that although these studies use different methodologies, the confusability matrices are highly similar with the only exception of pain confused with fear in Wang’s study^19^. These patterns of results therefore suggest that employing strategies to modulate spatial frequencies or facial regions^14^ availability offer similar results in terms of facial expression confusions as when only broadband faces are used^19^. Another interesting result from the confusability matrices is that happiness is systematically confused with pain in all three studies. This is consistent with Roy’s findings^21^ suggesting that there is an overlap in the information signaling joy and pain facial expressions in pain facial expressions.

 Furthermore, our results revealed that categorizing pain among other basic emotions is more accurate when stimuli are in a distance range of 1.2–4.8 m from the observer rendering available a broad range of object based SFs from low to high. The important decrease in performance between 4.8 and 9.6 m emphasizes the crucial role of MSFs between 16 and 32 cpf. Taken together, the results from the SF Bubbles method and the distance experiment highlight the importance of MSF in pain recognition, although an advantage for LSF is found when solely comparing LSF to HSF tuning.

In addition to being associated with arbitrary decisions, the utilization of cutoffs present an important downfall by hindering the possible contribution of a large band of MSF, which, as revealed in this study, are diagnostic for the recognition of pain facial expressions. Indeed, cutoff methods used in previous research hide the complexity of SF information utilization and lead to misleading conclusions. Even though we found consistent results when using the same criterion as past studies for cutoffs of LSF and HSF (i.e. LSFs were more useful than HSFs), this does not mean that LSFs are the most useful SFs for pain facial expression decoding. Needless to say, Wang’s^19^ study is by no means the only one to use arbitrary cutoffs to separate HSF and LSF (e.g.^22,24,25^) that are not theoretically driven but rather informed by methodological issues (e.g.^26,27^ or by concerns for the replication of previous studies^28^. Furthermore, given the central role of MSF in many face perception tasks such as identification^30,36,42,43^ and facial expression categorization^44^ it is substantial to include them in tasks investigating pain recognition or any other facial expressions. Therefore, it is critical to use methods and experimental paradigms that allow us to investigate the full SF spectrum. SF Bubbles method is effective but other methods such as critical band masking^42,45^ and bandpass filtering^46^ are also suitable. Since several existing methods allow us to assess the contribution of each SF to performance, it would be crucial that future studies make use of such methods and avoid arbitrary cutoffs of low and high SFs.

Even if facial expression is one of the most effective ways to express pain^2^, other non-verbal cues could be useful for pain recognition. Considering that pain signals expressed through facial expressions are difficult to transmit over long distances and could be confused with other facial expressions, studies addressing screams or cries of pain are of great interest. One could also consider circumstances where healthcare professionals in hospitals have to react to pain signals expressed by vocalization when the patients’ face is not nearby or even discriminate between screams of pain from anger. Interestingly, a recent study on speech prosodies argues that recognition of emotional expression is better understood as gradients representing blends of emotions rather than distinct categories of emotions (e.g. anger or pain)^47^. Their work suggests that pain recognition relies mostly on two emotional prosody dimensions (i.e. distress and sadness) that are preserved across cultures. These results are interesting considering that facial expressions of sadness and pain are often confused^14,19^. To our knowledge, no study has clearly investigated the impact of distance on pain facial expression recognition when combined with pain vocalizations. One would expect that concurrent vocal signals of pain would facilitate quicker reaction times for facial pain recognition and better discrimination between different facial expressions that could be confused with pain (e.g. disgust and sadness).

Although only white Canadian participants were tested in this study, it is interesting to discuss how these data might be relevant to other cultures considering that pain seems to be expressed with similar sets of facial features^48,49^ suggesting the existence of universal facial expressions of pain. In this case, one would expect that the distance at which one recognizes an expression of pain would be similar across cultures and would also rely on the same range of spatial frequencies. On the other hand, cross-cultural studies on face perception have uncovered cultural differences in visual processes as early as spatial frequency extraction^29,37^. Studies regarding pain and other basic facial expressions have also suggested that culture modulates the visual strategies (i.e. eye movements and mental representations) underlying facial expression recognition^50–52^. Although facial expressions of pain may be expressed similarly in terms of facial features, one difference that seems important to consider is the intensity with which pain is expressed across cultures^53^. As a matter of fact, differences in the intensity of pain expression appear to have an impact on the ability to decode pain in another cultural group^53^. Taking into account these recent data on cross-cultural differences, it seems clear that further research on spatial frequency tuning for pain recognition across various cultures is needed.

There are some limitations and interesting future directions to be considered. Firstly, one potential limit of the study concerns the choice of stimuli. The stimuli used here came from a validated database^54,55^ composed of photos of 10 different identities successively expressing one of seven emotions (i.e. anger, disgust, fear, joy, sadness, surprise and pain) at a comparable, strong intensity level or displaying a neutral expression. These stimuli consist of facial expressions produced on request (i.e. posed facial expressions) as opposed to spontaneously induced expressions. One could argue that the latter are more ecologically valid and could potentially lead to different results than those obtained in the present study. Interestingly, a group of researchers^56^ has shown a high degree of similarity in visual strategies when posed and spontaneous facial expressions are compared. However, they do reveal that there is a higher degree of heterogeneity in the useful facial cues across identities in spontaneous expressions^56^. This implies that different facial features could be more/less useful for different expressors. Given these results, it would be interesting for future studies to compare posed and spontaneous pain facial expressions and to verify whether SF tuning differs across these conditions. It would also be interesting to investigate visual information extraction in terms of facial cues as in Roy and colleagues’ study^14^ with the Bubbles method but with spontaneous pain facial expressions. Secondly, another potential limit of this study with respect to our choice of stimuli concerns their static display. In everyday life, facial expressions are generated with facial movement and are dynamics. Hence, a more ecological way of investigating pain facial expression recognition would be to use dynamic stimuli. Interestingly, it has been shown that a slight shift toward lower SFs occurs for dynamic expressions (i.e. anger, disgust, fear, joy, sadness, surprise) in comparison with static ones^57^. These authors suggest that it is the presence of motion in general that causes this shift in SFs and not motion information per se*,* since a shift to LSFs is also observed when frames of dynamic facial expressions are shuffled. Considering the broad literature supporting the advantage of dynamic facial expression over static ones (e.g.^57,58^) and the shift toward lower SFs in their processing, it would be interesting to replicate these findings using dynamic pain facial expression.

Altogether, this study not only reconciles data from different groups of researchers, but reveals the SFs useful for pain recognition through different methods. Indeed, results with SF Bubbles are corroborated by another experiment using a completely different methodology (i.e. distance experiment). The convergence of these findings is interesting since it enables greater extrapolation of our results to real life situations that may potentially be tied to evolutionary hypotheses. For example, experiment 2 revealed that pain categorization is more accurate and mostly identical between a perceived distance of 1.2 to about 4.8 m which corresponds to all conditions in which all MSFs and LSFs are available. These data are particularly interesting in the context of healthcare, where time and resources may not always be available even though a rapid and accurate assessment of the presence of pain experienced by patients is crucial. These results suggest that healthcare providers could recognize the presence of pain even from the door frame of a patient’s room if it is no more than about 5 m away. Furthermore, this perceptual treatment is compatible with survival mechanisms since in less than a few seconds (i.e. a distance of a few meters), a person in pain will receive assistance.

## Conclusion

In sum, this study revealed through two complementary experiments the SF information involved in pain recognition. The results reconcile previous data from different groups of researchers, and highlight the importance of a broad range of SFs starting from low to relatively high. Most importantly, these findings stress the importance of MSFs in pain recognition and suggest that any method that removes these SFs does not provide a true portrait of visual processing of pain.

## Methods

### Participants

Twenty healthy adult participants between 18 and 40 years old took part in experiment 1 (10 women; mean age of 26 years-old; SD = 3.4) and another twenty participants took part in experiment 2 (14 women; mean age of 21.45 years-old; SD = 3.52). All participants identified as white Canadians. The sample size for all experiments was chosen based on similar studies (between 3 and 28; e.g.^29,36,57^). According to recent studies using the SF Bubble’s method^30,34^, the results obtained with this method are generally very robust since they are based on many trials for a single task. For both experiments, participants provided their written and informed consent for participating in the experiments. They all had normal or corrected-to-normal vision as indicated by their score on the Snellen Chart and Pelli-Robson Contrast Sensitivity Chart^59^ and were compensated 12$/hour for their participation. All procedures were carried out with the ethics approval of the Université du Québec en Outaouais and all experiments conformed to relevant guidelines and regulations with regard to the use of human participants.

### Material and stimuli

The stimuli came from the STOIC validated database^54,55^ composed of photos of 10 different white identities expressing seven emotions (i.e. anger, disgust, fear, joy, sadness, surprise and pain) at a strong, comparable intensity level or displaying a neutral expression for a total of 80 unique faces. These stimuli are the same as the ones used by Wang and coll.^19,20^ and Roy and coll.^14,21^. They were gray-scaled and their global orientation was normalized. All stimuli were equated in their mean luminance, contrast and SF spectrum using the SHINE toolbox for Matlab^60^. A grey mask with an elliptic hole was applied to each face to hide the hair and ears of the stimuli as well as the background. Note that informed consent for publication of identifying images in an online open-access publication was obtained for all face stimuli presented in this article.

Stimuli were displayed on a calibrated LCD monitor with a resolution of 1080p and a refresh rate of 100 Hz. The experimental program was written in Matlab (Natick, MA), using functions from the Psychophysics Toolbox^59,61^. The face width subtended 5.72 degrees of visual angle and the viewing distance was maintained at 46.5 cm using a chinrest for the first experiment and 122 cm for the second experiment.

#### Manipulation of spatial frequencies: Bubble’s method

To reveal the visual information useful for the recognition of facial expressions the SF Bubble’s method was used^30,36^ in the first experiment. The method consists of randomly sampling the visual information contained in a stimulus on a trial-by-trial basis, such that a different subset of this information is rendered available to the participant. For example, in one trial, only MSF may have been sampled while on the next trial both LSF and HSF may have been presented to the participant. Across trials, all combinations of SF were therefore possible. The correlation of the participant’s performance on a trial-by-trial basis with the availability of each SF can then be calculated.

**Figure 5:**
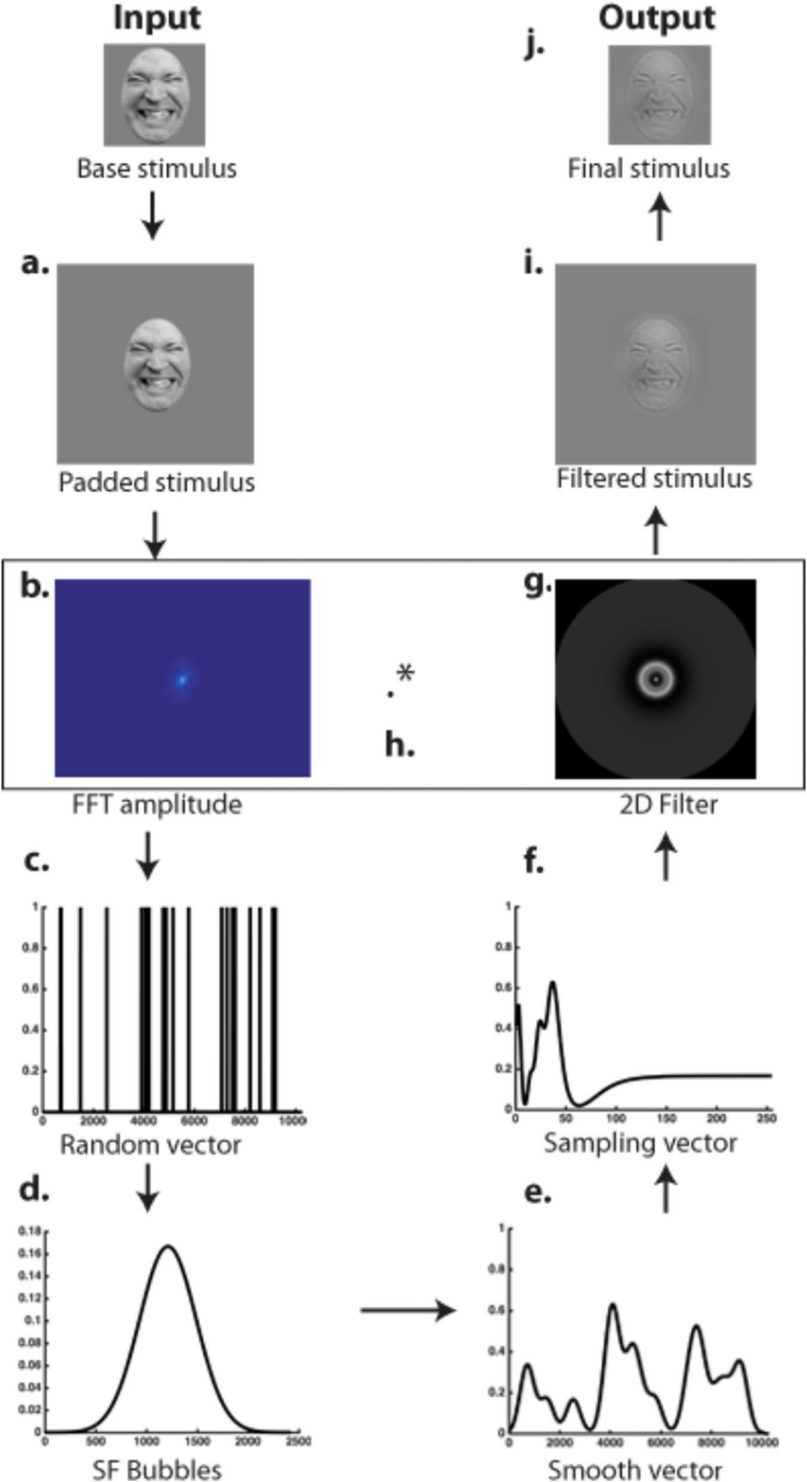
Creation of a bubblized stimulus using the SF Bubble’s technique (see text for details).

The creation of a stimulus with SF Bubbles went as follows (see Fig. 5; for more details about the SF Bubble’s method, see^30^). First, the base stimulus was padded with a uniform gray background of twice the stimulus size in order to minimize edge artifacts in the SF domain. Second, the padded stimulus was fast Fourier transformed (FFT) using functions from the Image Processing Toolbox for MATLAB (Natick, MA). To create a random SF filter, a binary random vector of 2*wk* elements was generated (c), where *w* was the stimulus width (256 pixels) and *k* a constant that determined the smoothness of the sampling; *k* was set to 20 for all the experiments reported in this article. The random vector thus had 10,240 elements. The vector contained zeros among which *b* ones were randomly distributed (with replacement). Parameter *b* thus determined the number of SF bubbles and was set to 10. To create a smooth filter, the binary vector was convolved with a Gaussian kernel, referred to as a SF bubble (d). The standard deviation of the SF bubble was set to 1.8 cycles per image. The convolution resulted in a “sampling vector” consisting of *b* randomly located SF bubbles (e). This smoothed vector was then subjected to a logarithmic transformation (f) in order to fit the human visual system’s SF sensitivity^62^. The resulting *w*-element filter was then rotated about its origin to create an isotropic random two-dimensional filter of size *w* × *w* (g). Filtering was carried out by dot-multiplying the two-dimensional filter with the complex amplitude of the padded base stimulus before subjecting the result to the inverse Fourier transform. We constructed the experimental stimuli by cropping the central *w* × *w* pixel region of the filtered image.

#### Manipulation of spatial frequencies: distance

In order to examine whether the results obtained with the Bubble’s method are consistent with a more ecological method, participants completed a distance task where the distance at which a facial expression can be perceived was manipulated. This method is inspired by the work of Smith and Schyns^16^ who investigated the effectiveness of the transmission of emotion signals over different viewing distances. Here we used this method to reveal how pain facial expressions can be recognized across various distances and therefore at which SF. Indeed, increasing perceived distance between a stimulus and an observer represents an ecological way to manipulate SF since it decreases the availability of higher SFs. We first created stimuli using the Laplacian Pyramid toolbox^63^, a method that recursively removes the highest SFs of an image while down-sampling the residual image by a factor of two in order to create six reduced-size images simulating increasing viewing distances (see Fig. 4). We used the Laplacian Pyramid because it removes one octave of SF between images of different sizes, which corresponds roughly to a similar decrement in spectral energy. Note that there was no loss of face SF information from the filtered original image to the reduced size image, despite the reduction in size. The original image size was 384 × 384 pixels (~ 6.9 cm), which corresponds to 3.26 degrees of visual angle. The impact of distance on SF information acts as a low-pass filter, where the original image contains available SFs (i.e. 128 cpf) and subsequent images respectively contains information under 64 cpf, 32 cpf, 16 cpf, 8 cpf and 4 cpf. The simulated viewing distances corresponded to 1.2, 2.4, 4.8, 9.6, 19.2, and 38.4 m (or respectively 3.26, 1.63, 0.815, 0.41, 0.20, 0.10 degree of visual angle).

### Procedure

Prior to both experiments, participants were invited to look at the emotional faces displayed on a computer screen. When they felt confident that they could recognize all facial expressions, a practice session began. Each trial began with a fixation cross displayed in the center of the screen for 500 ms. One of the 80 stimuli was then randomly selected and presented for 300 ms. The next trial began right after the participant’s response. Participants responded by pressing one of the eight keyboard keys associated with each emotion. No response time limit was imposed and no accuracy feedback was provided. The main goal of the learning phase was to ensure that participants were able to recognize each facial expression. The learning phase was completed when performance was above 90% correct for two consecutive blocks of 160 trials. Participants then completed either experiment 1 or 2. The only difference between both experiments is that faces filtered with SF bubbles were presented to participants in experiment 1 and faces varying in sizes were presented in experiment 2. The same procedure as in the practice session was conducted for both experiments. A small amount of white Gaussian noise was added to the stimulus, with the amount of noise manipulated on a trial-by-trial basis using QUEST^64^ to maintain performance halfway between chance and perfect performance. Participants completed 26 blocks for a total of 4160 trials (each face was repeated 52 times) per participant in experiment 1 and 15 blocks for a total of 2400 (each face was repeated 30 times) trials per participant in experiment 2. Of course, participants completed the experiments in several sessions and took breaks as needed.

## Supplementary Information

Supplementary Tables.

## Data Availability

The datasets generated during and/or analyzed during the current study are available in the OSF repository, https://osf.io/cn9ed/?view_only=6d684d4cf9524c6dbb2fad0fd3643138.
