# Supplementary material for: The role of spatial frequencies for facial pain categorization
Source: Sci Rep. 2021 Jul 13;11:14357. doi: 10.1038/s41598-021-93776-7 (PMC8277883; doi:10.1038/s41598-021-93776-7)
Supplement: Supplementary file 1 — Supplementary Tables. [file 41598_2021_93776_MOESM1_ESM.docx]

Supplementary Tables

Supplementary Table S1 Confusability matrix depicting the proportion of responses (columns) for each target emotion presented (rows) for a simulated distance of 1.2 meters.

|  | Emotion presented |  |  |  |  |  |  |  |
| --- | --- | --- | --- | --- | --- | --- | --- | --- |
| Emotion perceived | Pain | Disgust | Fear | Happy | Neutral | Anger | Sadness | Surprise |
| Pain | **0.807(0.6728)** | 0.056 | 0.019 | 0.028 | 0.006 | 0.017 | 0.056 | 0.011 |
| Disgust | 0.071 | **0.803(0.6731)** | 0.022 | 0.011 | 0.015 | 0.031 | 0.036 | 0.011 |
| Fear | 0.008 | 0.022 | **0.819(0.6741)** | 0.012 | 0.002 | 0.008 | 0.012 | 0.117 |
| Happy | 0.003 | 0.002 | 0.004 | **0.96(0.8719)** | 0.02 | 0.005 | 0.002 | 0.004 |
| Neutral | 0.023 | 0.035 | 0.012 | 0.023 | **0.827(0.6874)** | 0.007 | 0.046 | 0.027 |
| Anger | 0.007 | 0.019 | 0 | 0.011 | 0.017 | **0.928(0.8569)** | 0.011 | 0.007 |
| Sadness | 0.047 | 0.015 | 0.017 | 0.008 | 0.078 | 0.008 | **0.806(0.6656)** | 0.021 |
| Surprise | 0.002 | 0.006 | 0.102 | 0.004 | 0.03 | 0.001 | 0.007 | **0.848(0.6875)** |

Hits are presented in the diagonal in bold, unbiased hits between parentheses, while omissions (rows – regular font) and false alarms (columns – regular font) are reported for each emotion in the rest of the matrix.

Supplementary Table S2 Confusability matrix depicting the proportion of responses (columns) for each target emotion presented (rows) for a simulated distance of 2.4 meters.

|  | Emotion presented |  |  |  |  |  |  |  |
| --- | --- | --- | --- | --- | --- | --- | --- | --- |
| Emotion perceived | Pain | Disgust | Fear | Happy | Neutral | Anger | Sadness | Surprise |
| Pain | **0.822(0.6825)** | 0.05 | 0.016 | 0.013 | 0.005 | 0.015 | 0.064 | 0.015 |
| Disgust | 0.081 | **0.836(0.6989)** | 0.014 | 0.001 | 0.014 | 0.015 | 0.032 | 0.007 |
| Fear | 0.006 | 0.032 | **0.795(0.6625)** | 0.008 | 0.005 | 0.005 | 0.016 | 0.133 |
| Happy | 0 | 0.003 | 0.01 | **0.968(0.9053)** | 0.011 | 0.003 | 0.001 | 0.004 |
| Neutral | 0.017 | 0.037 | 0.016 | 0.024 | **0.845(0.7014)** | 0.004 | 0.038 | 0.019 |
| Anger | 0.007 | 0.022 | 0.003 | 0.006 | 0.012 | **0.942(0.8972)** | 0.003 | 0.005 |
| Sadness | 0.052 | 0.018 | 0.016 | 0.012 | 0.102 | 0.005 | **0.773(0.6363)** | 0.022 |
| Surprise | 0.005 | 0.002 | 0.084 | 0.003 | 0.024 | 0 | 0.012 | **0.87(0.015)** |

Hits are presented in the diagonal in bold, unbiased hits between parentheses, while omissions (rows – regular font) and false alarms (columns – regular font) are reported for each emotion in the rest of the matrix.

Supplementary Table S3 Confusability matrix depicting the proportion of responses (columns) for each target emotion presented (rows) for a simulated distance of 4.8 meters.

|  | Emotion presented |  |  |  |  |  |  |  |
| --- | --- | --- | --- | --- | --- | --- | --- | --- |
| Emotion perceived | Pain | Disgust | Fear | Happy | Neutral | Anger | Sadness | Surprise |
| Pain | **0.796(0.6472)** | 0.037 | 0.023 | 0.027 | 0.006 | 0.021 | 0.078 | 0.012 |
| Disgust | 0.088 | **0.776(0.6341)** | 0.023 | 0.003 | 0.015 | 0.048 | 0.038 | 0.006 |
| Fear | 0.017 | 0.034 | **0.759(0.6122)** | 0.001 | 0.01 | 0.007 | 0.021 | 0.151 |
| Happy | 0.003 | 0.001 | 0.006 | **0.946(0.8887)** | 0.033 | 0.002 | 0.004 | 0.005 |
| Neutral | 0.022 | 0.039 | 0.03 | 0.015 | **0.754(0.5668)** | 0.011 | 0.075 | 0.054 |
| Anger | 0.008 | 0.048 | 0.004 | 0.006 | 0.022 | **0.901(0.8126)** | 0.008 | 0.003 |
| Sadness | 0.039 | 0.018 | 0.014 | 0.004 | 0.148 | 0.006 | **0.756(0.5773)** | 0.015 |
| Surprise | 0.006 | 0.001 | 0.082 | 0.005 | 0.015 | 0.003 | 0.01 | **0.878(0.6858)** |

Hits are presented in the diagonal in bold, unbiased hits between parentheses, while omissions (rows – regular font) and false alarms (columns – regular font) are reported for each emotion in the rest of the matrix.

Supplementary Table S4 Confusability matrix depicting the proportion of responses (columns) for each target emotion presented (rows) for a simulated distance of 9.6 meters.

|  | Emotion presented |  |  |  |  |  |  |  |
| --- | --- | --- | --- | --- | --- | --- | --- | --- |
| Emotion perceived | Pain | Disgust | Fear | Happy | Neutral | Anger | Sadness | Surprise |
| Pain | **0.594(0.4383)** | 0.055 | 0.033 | 0.135 | 0.017 | 0.079 | 0.061 | 0.026 |
| Disgust | 0.089 | **0.58(0.3823)** | 0.043 | 0.009 | 0.035 | 0.175 | 0.049 | 0.02 |
| Fear | 0.008 | 0.051 | **0.697(0.4627)** | 0.01 | 0.013 | 0.011 | 0.026 | 0.184 |
| Happy | 0.016 | 0.007 | 0.011 | **0.908(0.7329)** | 0.039 | 0.005 | 0.008 | 0.006 |
| Neutral | 0.018 | 0.05 | 0.059 | 0.037 | **0.597(0.3618)** | 0.042 | 0.112 | 0.085 |
| Anger | 0.027 | 0.088 | 0.023 | 0.007 | 0.066 | **0.747(0.5138)** | 0.017 | 0.025 |
| Sadness | 0.048 | 0.039 | 0.043 | 0.014 | 0.199 | 0.022 | **0.608(0.4149)** | 0.027 |
| Surprise | 0.005 | 0.01 | 0.141 | 0.005 | 0.019 | 0.005 | 0.01 | **0.805(0.5501)** |

Hits are presented in the diagonal in bold, unbiased hits between parentheses, while omissions (rows – regular font) and false alarms (columns – regular font) are reported for each emotion in the rest of the matrix.

Supplementary Table S5 Confusability matrix depicting the proportion of responses (columns) for each target emotion presented (rows) for a simulated distance of 19.2 meters.

|  | Emotion presented |  |  |  |  |  |  |  |
| --- | --- | --- | --- | --- | --- | --- | --- | --- |
| Emotion perceived | Pain | Disgust | Fear | Happy | Neutral | Anger | Sadness | Surprise |
| Pain | **0.269(0.0970)** | 0.075 | 0.093 | 0.279 | 0.052 | 0.076 | 0.081 | 0.072 |
| Disgust | 0.11 | **0.19(0.0565)** | 0.417 | 0.096 | 0.12 | 0.226 | 0.08 | 0.085 |
| Fear | 0.059 | 0.073 | **0.417(0.1456)** | 0.028 | 0.043 | 0.038 | 0.054 | 0.288 |
| Happy | 0.078 | 0.034 | 0.044 | **0.553(0.2603)** | 0.113 | 0.051 | 0.071 | 0.056 |
| Neutral | 0.059 | 0.062 | 0.114 | 0.076 | **0.322(0.1017)** | 0.087 | 0.112 | 0.168 |
| Anger | 0.063 | 0.107 | 0.101 | 0.075 | 0.128 | **0.328(0.1163)** | 0.072 | 0.126 |
| Sadness | 0.085 | 0.08 | 0.123 | 0.051 | 0.203 | 0.096 | **0.246(0.0811)** | 0.116 |
| Surprise | 0.023 | 0.018 | 0.206 | 0.017 | 0.039 | 0.023 | 0.03 | **0.644(0.2667)** |

Hits are presented in the diagonal in bold, unbiased hits between parentheses, while omissions (rows – regular font) and false alarms (columns – regular font) are reported for each emotion in the rest of the matrix.

Supplementary Table S6 Confusability matrix depicting the proportion of responses (columns) for each target emotion presented (rows) for a simulated distance of 38.4 meters.

|  | Emotion presented |  |  |  |  |  |  |  |
| --- | --- | --- | --- | --- | --- | --- | --- | --- |
| Emotion perceived | Pain | Disgust | Fear | Happy | Neutral | Anger | Sadness | Surprise |
| Pain | **0.099(0.0144)** | 0.102 | 0.149 | 0.103 | 0.164 | 0.076 | 0.113 | 0.194 |
| Disgust | 0.082 | **0.105(0.0153)** | 0.155 | 0.076 | 0.204 | 0.085 | 0.121 | 0.172 |
| Fear | 0.081 | 0.069 | **0.207(0.0358)** | 0.069 | 0.152 | 0.063 | 0.09 | 0.269 |
| Happy | 0.089 | 0.096 | 0.131 | **0.125(0.0219)** | 0.194 | 0.083 | 0.119 | 0.163 |
| Neutral | 0.071 | 0.107 | 0.119 | 0.093 | **0.213(0.0315)** | 0.062 | 0.116 | 0.219 |
| Anger | 0.089 | 0.079 | 0.138 | 0.096 | 0.217 | **0.077(0,0104)** | 0.124 | 0.197 |
| Sadness | 0.095 | 0.087 | 0.12 | 0.088 | 0.096 | 0.077 | **0.115(0.0146)** | 0.201 |
| Surprise | 0.073 | 0.076 | 0.177 | 0.064 | 0.164 | 0.049 | 0.107 | **0.358(0.0723)** |

Hits are presented in the diagonal in bold, unbiased hits between parentheses, while omissions (rows – regular font) and false alarms (columns – regular font) are reported for each emotion in the rest of the matrix.
